# Safety and Performance of POLYTECH Mesmo Breast Implants: A 5-Year Post-market Surveillance Study on 919 Patients

**DOI:** 10.1093/asjof/ojac011

**Published:** 2022-02-07

**Authors:** Paolo Montemurro, Giacomo Siri, Luana Clerico

**Affiliations:** Department of Mathematics, University of Genoa, Genoa, Italy; Clinical and Medical Affairs Department, Polytech Health & Aesthetics, Dieburg, Germany

## Abstract

**Background:**

In 2007, POLYTECH Health & Aesthetics (POLYTECH, Dieburg, Germany) established an ongoing patient survey to improve the post-market surveillance of silicone gel-filled breast implants based on patient-reported outcomes in the context of the pioneering “Implants of Excellence” (IoE) program.

**Objectives:**

To disclose an update on safety and performance outcomes at 5 years for Mesmo breast implants.

**Methods:**

Between January 2014 and October 2019, 919 patients (for a total of 1816 implants) who underwent breast augmentation and reconstruction with Mesmo implants were asked to participate in the IoE program. Data were collected by mean of 1320 questionnaires received. A survival analysis assessed the onset of different complications.

**Results:**

Eight patients (0.9%) experienced capsular contracture Baker grade III or IV with a cumulative rate at 5 years of 1.2% (95% CI = 0.6-2.4). The proportion of revisional surgery was 0.5% with a 5-year rate of 0.6% (95% CI = 0.2-1.5). Additional adverse events such as hematoma, seroma, malposition, open wounds, and other complications were carefully monitored. Questionnaires showed that 93.9% (95% CI = 92.2-95.4) of the patients were satisfied or very satisfied with their aesthetic results with Mesmo implants.

**Conclusions:**

Post-market clinical follow-up revealed that the overall complications rate reported was low. Data demonstrated an excellent safety property on a large cohort of patients. This result allows the rating of Mesmo breast implants as highly competitive and a very safe choice for both surgeons and patients.

**Level of Evidence: 3:**

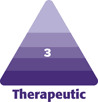

Since the introduction of breast implants in plastic surgery in the early 1960s,^1^ these devices have undergone a significant evolution with modifications in the composition, surface, and shape.^2-4^ Various types of implants are available today and they are generally divided according to their shape (round or anatomically shaped), their surface (usually either smooth or textured), and the material used to fill them (saline or silicone).^5^ Currently, high-cohesive silicone gel implants are the most commonly used devices for aesthetic and reconstructive breast surgery.^6^

Despite improvements in design and technology, implant-based breast augmentation and reconstruction are still associated with a number of risks and complications and commercially available breast implants differ in the way they interact with the soft tissues.^7^ The most common complication is capsular contracture (CC) that often leads to the need for a reoperation, as described in recent studies.^8-12^ CC is traditionally classified using the Baker system,^13^ a subjective classification based upon the physician’s clinical findings on the patient’s breast status.

A plastic surgeon’s aim is obviously to use and recommend implants that are associated with lower rates of complications,^14^ which is why it is crucial to gather as much information as possible regarding the safety and performance of breast implants in the long term. Therefore, in 2007, POLYTECH Health & Aesthetics, (POLYTECH, Dieburg, Germany), established a warranty program called “Implant of Excellence” (IoE). The aim of this program is to further improve the post-market surveillance (PMS) of breast implants and to increase the knowledge of real-world safety data. The IoE program involves all the patients who underwent breast surgery with implants made by POLYTECH and registered themselves through the portal.

The IoE design adheres to the latest indication of the post-market clinical follow-up (PMCF) of medical devices. The Medical Device Regulation (European Union) 2017/745 (MDR) considers the PMCF as a continuous process that updates the clinical evaluation and that shall be addressed in the manufacturer’s PMS plan. The aims of the PMCF plan are (1) confirming the safety and performance, including the clinical benefit if applicable, of the device throughout its expected lifetime; (2) identifying previously unknown side effects and monitoring the identified side effects and contraindications; (3) identifying and analyzing emergent risks on the basis of factual evidence; (4) ensuring the continued acceptability of the benefit-risk ratio; and (5) identifying possible systematic misuse or off-label use of the device, with a view to verifying that the intended purpose is correct (MDCG 2020-7).

Mesmo breast implants were introduced into the market in 2011. They are high-cohesive gel-filled implantable devices with the least rough-textured surface of the POLYTECH portfolio. The International Organization for Standardization 14607:2018, as the only official and shared classification method currently available, define as micro-textured the surfaces with an average roughness between 10 and 50 μm.^15^ Therefore, Mesmo implants, with a roughness of 25 μm, is classified, based on the average roughness measurement on the finished device, as a micro-textured surface.^16^

This paper describes the results of the systematic collection of all adverse events and the patient satisfaction scores related to Mesmo implants reported in the IoE questionnaires since 2014, when the database was updated to provide the data collection with a structure useful for proper statistical analysis. The present paper reports the 5-year results of this PMCF.

## METHODS

This study encompasses 919 consecutive patients who underwent breast surgery with Mesmo implants and registered themselves in the IoE program between January 2014 and March 2018. All of them were made aware of this warranty program by their treating surgeon. The procedures performed in this observational study were in accordance with the ethical standards of the IRB and with the 1964 Helsinki declaration and its later amendments. The first completed questionnaire was sent back and received in May 2015, and the last questionnaire in October 2019 (even if the cutoff point was fixed at the end of December 2019).

Undoubtedly, data collected by the physician give an unparalleled value to any study conducted to evaluate the incidence of complications after a given surgery. As a matter of fact, this study does not aim to try and substitute the physician judgment, which remains the gold standard. Nevertheless, it must be noted that patients-reported outcomes (PROs) are widely used and recognized as a valid mean to measure the overall efficacy and safety of a clinical intervention from the patients’ perspective.^17-19^ Determining a satisfactory patient outcome following breast surgery is a multi-faceted process involving patient education, informed consent process, surgical technique, and postoperative care.^20^

Of note, no distinction was made in the questionnaire between breast augmentation and breast reconstruction. There were no exclusion criteria, so even patients affected by other comorbidities (ie, autoimmune diseases and metabolic diseases) or other relevant demographic factors (ie, smoking habits, age, and BMI) could participate in the survey. This safety survey included patients from Europe, Commonwealth of Independent States countries (formerly countries of the Union of Soviet Socialist Republics), and Turkey, for a total of 43 countries.

Patients registered in the program were asked to complete a digital questionnaire every year (**Table 1**) regarding their clinical status from the date of surgery or from the last questionnaire sent. As an incentive, participating patients who answered yearly the questionnaire could benefit from an enlarged warranty coverage. Of note, given the scientific purpose of these data collection, all questionnaires received were analyzed, regardless of the yearly fulfillment of the enlarged warranty program dedicated to patients. The 919 enrolled patients provided POLYTECH with a total of 1320 questionnaires over the 5-years period of time of this study, with a mean response rate of 1.4 ± 0.8 questionnaires per patient (range, 1-5).

**Table 1.** Questionnaire Submitted to Patients

1. Does your breast/Do your breasts feel soft and natural?
2. Have you experienced any complications related to your breast implant/s since registering with the Implants of Excellence program/since receiving our last questionnaire?
3. What kind of complications related to your implant/s did occur?
a) Hematoma/e
b) Seroma/e
c) Hardening of the breast tissue
d) Implant dislocation
e) Open wounds
f) Other Please explain:
4. If you experienced unnatural breast-tissue hardening (see 3c), was it classified Baker Grade I, II, III, or IV?
a) Baker Grade I
b) Baker Grade II
c) Baker Grade III
d) Baker Grade IV
e) not classified
5. Were these complications ...
a) Short-term? (up to 3 months)
b) Long-term? (3 to 6 months)
c) Chronic? (more than 6 months)
6. Did you need surgical treatment due to these complications?
7. Was the implant concerned removed due to these complications?
8. Did your quality of life change?
9. Are you satisfied with the aesthetic result?
10. Would you decide again in favor of a breast augmentation/reconstruction with implants?

Baker Grade capsular contractures I and II are not clinically significant, because I describes a breast that looks and feels absolutely natural and II describes a breast with minimal contracture but no symptoms. Grades III and IV are clinically significant and symptomatic, with III describing moderate contracture with firmness felt by the patient, and IV describing severe contracture which is both obvious from observation and symptomatic for the patient.

Digital consent was provided, by which patients agreed to the use and analysis of their data. It should be noted that, if a complication covered by the warranty program occurs, the patient must provide POLYTECH with a detailed medical report together with photographs of the breast in order to obtain the due benefits. The questionnaire also included questions about the patient’s quality of life and satisfaction with the aesthetic outcome.

Data were analyzed by the biostatistician (G.S.) without distinction among different surgical techniques (eg, inframammary or periareolar incision, subglandular or submuscular implant placement). The evaluation focused on the status of the breasts, taking into consideration events such as revision surgery and implant removal or the occurrence of complications, such as CC Baker grades III and IV, malposition (referred to as “implant dislocation” in **Table 1**, answer 3d), hematoma, seroma, and wound-related healing problems. The rate of other, not better specified, complications (called “other” in **Table 1**, answer 3f) was also taken into consideration. All patients who reported revision surgery or implant removal (**Table 1**, answers 6 and 7) without having specified the reason for these surgeries were also considered as having “other” complications, in order to avoid an underestimation of the complications. Anyhow, as it follows from the setting of the questionnaire, participant patients were not asked to report the reason for revision surgery or implant removal.

Breakdown of complications by shape, profile, and volume was performed only for patients carrying the same implant (n = 906, 98.6%), either in both breasts (n = 884) or with 1 single implant (n = 22). Patients with 2 implants differing in shape, profile, or volume (n = 13) were excluded from this additional analysis.

**Figure 1.**
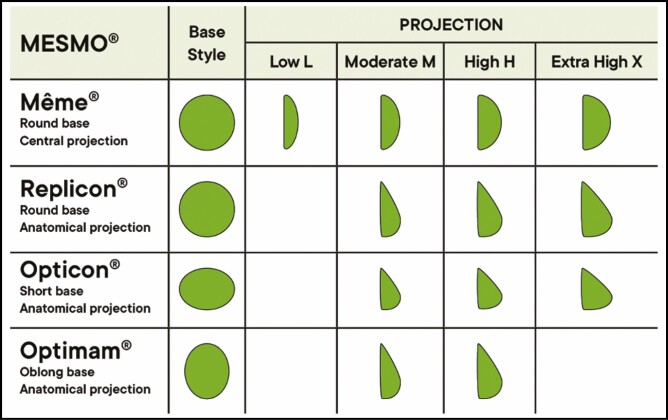
Mesmo POLYTECH SublimeLine (Dieburg, Germany). Même are round breast implants with a circular base and a uniformly convex profile; Replicon implants have a round base and a tear-drop profile with more volume in the lower half; Opticon anatomical implants have a shorter base; Optimam anatomical implants have an oblong base. The projection of these breast implants can be low, moderate, high, or extra high.

The main characteristics (base, style, and implant projection) of the different Mesmo implants monitored in this survey are shown in **Figure 1**.

### Statistical Analyses

All the analyses were performed at a patient level, not at a breast implant level. Continuous data were described in terms of mean and standard deviation, whereas categorical data were described in terms of counts and frequencies. A complication was defined as the onset in at least 1 of the 2 breasts. In the descriptive analysis, the associations between each type of complication and shape, profile and volume, were explored by contingency tables and Fisher’s Exact test was adopted. Kaplan-Meier analysis was used to assess the time to first complications, CC Baker grades III and IV, revisional surgery, and implant removal. The follow-up time was calculated in a conservative way to avoid an underestimation of the total rate of complications. Each patient was followed from the date of implant insertion to the last available questionnaire. In the case of implant removal, this date was considered as the last contact. The level of type I error was set to 5%. All analyses were performed with STATA 14 statistical software (version 14.2, StataCorp2015, College Station, TX).

## RESULTS

The present study includes 919 consecutive patients, with a total of 1816 Mesmo breast implants inserted from January 2014 to March 2018. The 5-year time frame for receiving questionaries ranged from January 2015 to December 2019 (first questionnaire collected in May 2015, last contact dated October 2019). The mean response rate per patient was of 1.4 ± 0.8 questionnaires (range, 1-5). Patients were followed for a mean period of 2.5 years (standard deviation [SD] = 1.1; range, 1.1-5.4). Three hundred and sixty-eight (40%) out of 919 patients were observed up to 3 years of follow-up, while 61 (6.6%) of them were followed up to 5 years. The mean volume of the breast implants inserted was 342.1 mL (SD = 69.2).

**Figure 2.**
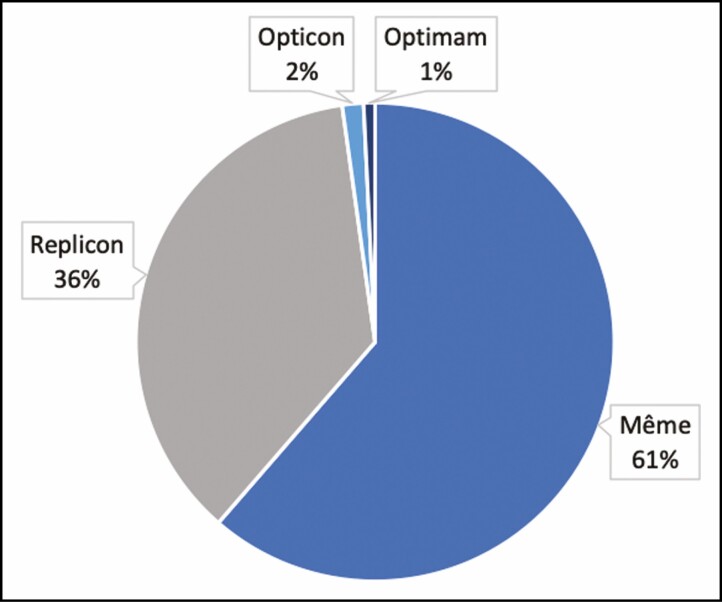
Distribution of Mesmo breast implants according to the shape.

The frequency of the different shapes for Mesmo breast implants used is shown in **Figure 2**. Most of the patients were operated with implants with a high profile (61.7%), 28.5% received implants with a moderate profile, and 9.8% carried implants with extra-high profile.

The statistical analysis of data reported by patients who registered themselves to the IoE program over time resulted in the identification of the principal adverse events that arose following the insertion of Mesmo breast implants.

**Figure 3.**
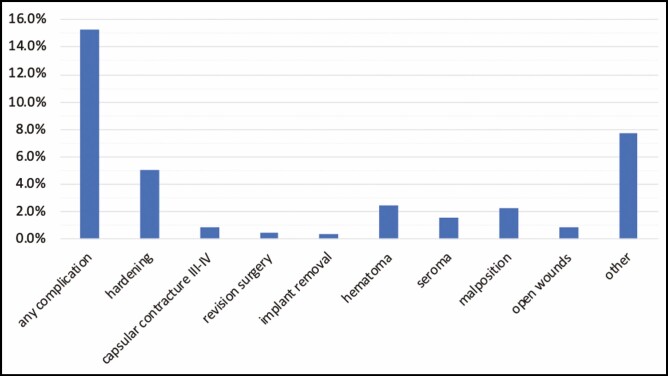
Proportion of patients (N = 919) with at least one complication in 1 of the 2 breasts during the follow-up period.

A survival analysis assessed that out of 919 patients, 141 (15.3%) experienced at least 1 complication among those listed in **Table 2** in at least 1 of the 2 breasts. **Figure 3** shows the proportion of patients with at least 1 complication in 1 of the 2 breasts during the follow-up period.

**Table 2.** Number of Patients by Complications, Overall and by Implant Shape

Complications, n (%)	Overall 919 (100%)	Round 556 (61.4%)	Anatomical 350 (38.6%)
Any complication *P* = 0.130	141 (15.3%)	77 (13.9%)	62 (17.7%)
Hardening (CC I-II) *P* = 0.062	47 (5.1%)	22 (4.0%)	24 (6.9%)
Capsular contracture III-IV *P* = 0.161	8 (0.9%)	7 (1.3%)	1 (0.3%)
Revision surgery *P* = 1.0	5 (0.5%)	3 (0.5%)	2 (0.6%)
Implant removal *P* = 0.642	4 (0.4%)	2 (0.4%)	2 (0.6%)
Hematoma *P* = 0.390	23 (2.5%)	12 (2.2%)	11 (3.1%)
Seroma *P* = 0.793	15 (1.6%)	10 (1.8%)	5 (1.4%)
Malposition *P* = 0.110	21 (2.3%)	9 (1.6%)	12 (3.4%)
Open wounds *P* = 0.718	8 (0.9%)	6 (1.1%)	2 (0.6%)
Other *P* = 0.122	71 (7.7%)	36 (6.5%)	33 (9.4%)

Complication is referred to as the onset of at least one of the events in at least 1 of 2 breasts for each patient. CC, capsular contracture.

There were 47 cases (5.1%) of hardening of the breast, a non-pathological condition also defined as CC Baker grade I or II. Eight patients (0.9% of the overall cases) experienced a severe CC Baker grade III or IV. The time to this complication ranged from 7.4 to 29.7 months with a mean time to the onset of 16.1 months (SD = 7.7). There were 5 patients (0.5%) who underwent revision surgery, and 4 (0.4%) who underwent implant removal. A total of 23 cases (2.5%) of hematoma and 15 cases (1.6%) of seroma were collected. It must be noted that the first case of seroma occurred 6.9 months after the surgery, and that the mean time to seroma was 18.3 months (SD = 12.0). Twenty-one cases (2.3%) of implant malposition and 8 cases (0.9%) of open wounds were recorded. Lastly, all complications that did not match the events described above were reported by patients as “other”: they accounted for a total of 71, with a proportion of 7.7%. Interestingly, the shape of the implants (round or anatomical) did not determine any statistically significant difference in the occurrence of complications (*P* = 0.130) (**Table 2**).

Implant profile used in the cohort surveyed were Moderate, High, or Extra high (**Table 3**). The Extra-high profile showed a higher risk for any complication compared with High and Moderate profiles (25.8%, 15.2%, and 12%, respectively; *P* = 0.011), especially for a prevalence of cases of hematoma (7.9%, 1.6%, and 2.7%, respectively; *P* = 0.006) and implant malposition (4.5%, 2.9%, and 0.4%, respectively; *P* = 0.014).

**Table 3.** Number of Patients by Complications, Overall and by Implant Profile

Complications, n (%)	Overall 919 (100%)	Moderate 258 (28.5%)	High 559 (61.7%)	Extra high 89 (9.8%)
Any complication* *P* = 0.011	141 (15.3%)	31 (12.0%)	85 (15.2%)	23 (25.8%)
Hardening (CC I-II) *P* = 0.058	47 (5.1%)	9 (3.5%)	28 (5.0%)	9 (10.1%)
Capsular contracture III-IV *P* = 0.381	8 (0.9%)	4 (1.6%)	4 (0.7%)	0 (0.0%)
Revision surgery *P* = 0.794	5 (0.5%)	2 (0.8%)	3 (0.5%)	0 (0.0%)
Implant removal *P* = 1.0	4 (0.4%)	1 (0.4%)	3 (0.5%)	0 (0.0%)
Hematoma* *P* = 0.006	23 (2.5%)	7 (2.7%)	9 (1.6%)	7 (7.9%)
Seroma *P* = 0.098	15 (1.6%)	3 (1.2%)	8 (1.4%)	4 (4.5%)
Malposition* *P* = 0.014	21 (2.3%)	1 (0.4%)	16 (2.9%)	4 (4.5%)
Open wounds *P* = 0.225	8 (0.9%)	1 (0.4%)	5 (0.9%)	2 (2.3%)
Other *P* = 0.069	71 (7.7%)	15 (5.8%)	42 (7.5%)	12 (13.5%)

Complication is referred to as the onset of at least one of the events in at least 1 of 2 breasts for each patient. CC, capsular contracture. *Statistically significant at 5%.

Complications were also analyzed in relation to breast implant volume. Implants with a volume of less than 350 mL were defined as low-volume implants, whereas implants with a volume of more than 350 mL were defined as high-volume implants (**Table 4).** In any case, the volume did not affect the occurrence of complications at all (*P* = 0.637).

**Table 4.** Number of Patients by Complications, Overall and by Implant Volume

Complications, n (%)	Overall 919 (100%)	Volume ≤ 350 553 (61.0%)	Volume > 350 353 (39.0%)
Any complication *P* = 0.637	141 (15.3%)	82 (14.8%)	57 (16.2%)
Hardening (CC I-II) *P* = 0.642	47 (5.1%)	30 (5.4%)	16 (4.5%)
Capsular contracture III-IV *P* = 0.494	8 (0.9%)	6 (1.1%)	2 (0.6%)
Revision surgery *P* = 0.163	5 (0.5%)	5 (0.9%)	0 (0.0%)
Implant removal *P* = 1.000	4 (0.4%)	3 (0.5%)	1 (0.3%)
Hematoma *P* = 0.393	23 (2.5%)	12 (2.2%)	11 (3.1%)
Seroma *P* = 1.000	15 (1.6%)	9 (1.6%)	6 (1.7%)
Malposition *P* = 0.258	21 (2.3%)	10 (1.8%)	11 (3.1%)
Open wounds *P* = 1.000	8 (0.9%)	5 (0.9%)	3 (0.9%)
Other *P* = 0.443	71 (7.7%)	39 (7.1%)	30 (8.5%)

Complication is referred to as the onset of at least one of the events in at least 1 of 2 breasts for each patient. CC, capsular contracture.

**Table 5** describes the Kaplan-Meier complication rates at 3 and 5 years. Analyzing the time to any complication, the Kaplan-Meier rate at 3 and 5 years for Mesmo implants was 17.8% (95% CI = 15.1-21.0) and 21.7% (95% CI = 18.0-26.1), respectively. The Kaplan-Meier rate of revision surgery at 3 and 5 years was 0.6% (95% CI = 0.2-1.5). The 3 and 5-year rate of implant removal was 0.4% (95% CI = 0.2-1.2). Analyzing the onset of CC Baker grades III-IV, the incidence rate at 3 and 5 years was 1.2% (95% CI = 0.6-2.4).

**Table 5.** Kaplan-Meier Complication Rates at 3 and 5 Years

Type of complication		Kaplan-Meier rate (95% CI)
Any complication	3	17.8% (15.1-21.0)
	5	21.7% (18.0-26.1)
Revision surgery	3	0.6% (0.2-1.5)
	5	0.6% (0.2-1.5)
Implant removal	3	0.4% (0.2-1.2)
	5	0.4% (0.2-1.2)
CC (Baker grades III-IV)	3	1.2% (0.6-2.4)
	5	1.2% (0.6-2.4)

The mean follow-up was of 2.5 ± 1.1 years. Out of 919 patients, 368 (40.0%) were observed up to 3 years of follow-up, and 61 (6.6%) up to 5 years. CC, capsular contracture.

**Figure 4.**
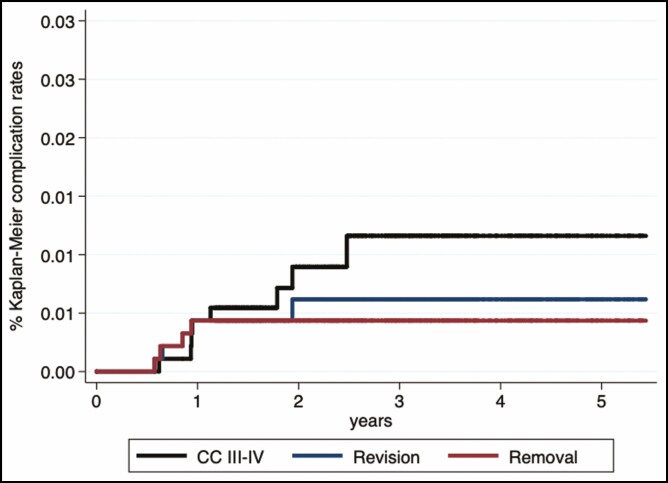
Kaplan-Meier curves for complications rates within a time frame of 5 years.

These data show that the 5-year probability for the patient of remaining free from CC Baker grades III and IV is 98.8% (95% CI = 97.6-99.4); the 5-year probability of remaining free from revision surgery is 99.4% (95% CI = 98.5-99.8); and the 5-year probability of remaining free from implant removal is 99.6% (95% CI = 98.8-99.8). The cumulative incidence functions for each type of complications are shown in Figure 4.

**Table 6** compares the main findings for Mesmo implants obtained in the present analysis with data published within the “core studies” for implants by the US American manufacturers having similar follow-up times and sample size, but different scientific approaches being data from the latter studies reported by physicians.^21,22^This comparison is merely descriptive as we appreciate the impossibility of a real one-to-one comparison between data collected by the patient and data collected by the physician. In addition, it has to be noted that in the “core studies” data were analyzed separately for breast augmentation and breast reconstruction.

**Table 6.** Comparison of Complication Rates Among Different Producers

Complications rates (%) at 5 years	MESMO all types of surgery N = 919	Textured Allergan P040046		Textured Mentor P060028	
		Primary augmentation N = 492	Primary reconstruction N = 225	Primary augmentation N = 572	Primary reconstruction N = 191
Any complication	21.7%	24.6%	46.6%	41.8%	59.1%
Revison surgery	0.6%	16.7%	39.4%	17.2%	41.2%
Implant removal	0.4%	8.2%	22.8%	6.0%	21.2%
CC (Baker grades III-IV)	1.2%	3.3%	9.6%	1.8%	9.5%

5-Year data. CC, capsular contracture.

The IoE survey also submitted questions to patients regarding their perception of the aesthetic appearance of their breasts and their quality of life (**Table 7**). The analysis of these data led to the understanding that 89.3% (95% CI = 87.2-91.3) of patients felt that their breasts were soft and natural, and that 93.9% (95% CI = 92.2-95.4) of patients were satisfied or very satisfied with the aesthetic result obtained with the insertion of Mesmo implants. For 95.6% (95% CI = 94.1-96.9) of patients, the quality of life improved or did not change, and 74.8% (95% CI = 71.8-77.5) of them would decide again in favor of surgery with these implants. It has to be pointed out that in case of mismatched values among subsequent surveys, the worst imputation has been chosen.

**Table 7.** Aesthetic Result and Quality of Life Improvement

Aesthetic result and quality of life improvement		N (%)	95% CI
Do your breasts feel soft and natural?	No	98 (10.7)	8.7-12.8
	Yes	821 (89.3)	87.2-91.3
Are you satisfied with the aesthetic result?	Very satisfied	501 (54.5)	51.2-57.8
	Satisfied	362 (39.4)	36.2-42.6
	Not satisfied	56 (6.1)	4.6-7.8
Did your quality of life change?	Improved	636 (69.2)	66.1-72.2
	Not changed	243 (26.4)	23.6-29.4
	Decreased	40 (4.4)	3.1-5.9
Would you decide again in favor of a surgery with implants?	Yes	687 (74.8)	71.8-77.5
	Maybe	187 (20.4)	17.8-23.1
	No	45 (4.9)	3.6-6.5

## DISCUSSION

The outcomes depicted in this analysis originate from a patient-oriented survey, part of the ongoing medical devices PMCF, in which data are collected annually. Despite we appreciate that data collected by the physician remain the gold standard when evaluating the incidence of a complication, the importance of this PMS represents a valid complement to it. In fact, it allows to obtain a patient-oriented view, which is indeed valuable, especially after the most recent safety issues associated with breast implants that have drawn the attention of the scientific community and public opinion, such as the emerging disease known as breast implant associated-anaplastic large cell lymphoma (BIA-ALCL).^23,24^ POLYTECH, with a farsighted and cautious vision, anticipating what is now compulsory for Medical Devices manufacturers, established a long time ago a program dedicated to patients with the aim to monitor the most common complications arising after breast implant insertion, identify previously unknown side-effects, and gain enough data to publish significant findings. The most important goals of this continuous monitoring are to confirm device safety and clinical performance, ensure continued acceptability of identified risks, and detect emerging risks on the basis of factual evidence. The data collected during the PMCF provide a continuous cycle of feedback to help improve quality management, user experience, and patients’ quality of life. Moreover, a thorough investigation protracted over time allows the company to constantly enhance the portfolio of the manufactured implants. It is with this aim in mind that POLYTECH developed a new micro-texturing in 2011 (Memso surface).

Since the introduction of breast implants more than 50 years ago, the international literature has reported several adverse events associated with these devices.^11,12,25,26^ The present 5-year analysis from the IoE program revealed some important information. First of all, we found that the incidence of the most severe complications related to Memso breast implants is very low.

The occurrence of CC, the major cause of morbidity and frequent cause of reoperation following implant surgery, reaches incidences as high as 50% in the published literature covering different implants.^27^ In our analysis, with data collected by patients and hence not directly comparable to other studies, we observed a cumulative CC (Baker grades III and IV) incidence of 1.2% (95% CI = 0.6-2.4) at 5 years for Memso breast implants, which means that the 5-year probability for the patients of remaining free from this tricky complication is 98.8% (95% CI = 97.6-99.4). It is also recalled that the patients of our cohorts underwent not only breast augmentation but also revisional surgery and breast reconstruction, a condition that, if combined with radiation therapy, can most likely lead to a severe CC grade.^28-30^

The incidence of revisional surgery is also considered a crucial marker of safety. In fact, revisional surgery is common following breast surgery with implants and it is requested/needed by patients when the complications of breast implants lead to a loss in the cosmetic appearance or pose a risk for their health. Surgeons, through the analysis of published clinical studies, are likely to choose the breast implant that shows the lower incidence of complications and revisional surgery. The present study shows that this cumulative incidence, reported by patients after insertion of Mesmo implants, is very low (Kaplan Meier rate = 0.6%, 95% CI = 0.2-1.5) at 5 years, namely that each patient has a 5-year probability of remaining free from revision surgery of 99.4% (95% CI = 98.5-99.8). This result, based on PROs and, therefore, not directly comparable to data presented by other studies encompassing other implants,^21,22,25,31-33^ offers a good outlook on the safety of the Mesmo devices.

Continuing to take all the necessary precautions while comparing the data obtained from the POLYTECH PMS survey with data published on the “core studies,” ^21,22^ our study shows that even the occurrence of implant removal with Mesmo implants is very low. The Kaplan-Meier cumulative incidence at 5 years of implant removal for Mesmo implants was 0.4% (95% CI = 0.2-1.2); therefore, we can also say that the 5-year probability for the patient of avoiding a further surgery is 99.6% (95% CI = 98.8-99.8).

The number of patients who experienced a late seroma with Memso implants (1.6% of patients) did not pose a problem for the safety of these patients in long term. Among 919 patients analyzed for 5 years, we did not find one single case of BIA-ALCL reported in the session “other.”

All the adverse events not classified as a defined disease, combined with patients’ dissatisfaction that required a revisional surgery, were assembled under the category of “other.” Also, the proportion of this type of events (7.7%) depicts a low complication rate associated with the use of Mesmo implants.

Complications that arose after Mesmo implants insertion were also analyzed in relation to breast implant shape, profile, and volume. The anatomical shape did not increase the occurrence of any complications with respect to a round shape (*P* = 0.130), even regarding implant malposition (*P* = 0.110). This very interesting finding seems to be in line with data previously published by other important studies.^21,22,34^ It has been observed only an increase in the incidence of hematoma (*P* = 0.006) and implant malposition (*P* = 0.014) following the insertion of extra-high-profile implants, a profile however barely used in this setting.

Interestingly, high-volume Mesmo implants (more than 350 mL) did not show a higher incidence of complications compared with low-volume implants. In this regard, literature reports that, in order to reduce the complications rate associated with the use of nanotextured implants, it is necessary to choose implants with a lower volume (<350 mL).^35^

The IoE survey did not ignore patients’ opinions on their perception of the aesthetic result and improvement in quality of life following breast surgery with Mesmo implants. In general, the responses of patients enrolled in the program showed a high level of satisfaction, with very high percentages, such as 93.9% (95% CI = 92.2-95.4) of patients satisfied or very satisfied with the aesthetic result (question 9 in Tables 1, 7), and 89.3% (95% CI = 87.2-91.3) of patients for whom the feel of their breasts was soft and natural (question 1 in Tables 1, 7). These data are referred to a longer period and seem to have better outcomes compared with those obtained by similar surveys in published literature.^36-38^ For example, the study of Ng et al^38^ reported that 90.8% and 87.1% of patients somewhat satisfied or very satisfied with the shape of their breast in breast augmentation and breast reconstruction cohort, respectively (data to be compared with question 9 of Table 1 of the present work), and 93.3% and 83.6% of patients somewhat satisfied or very satisfied with the feel of the breasts in augmentation and reconstruction cohort, respectively (data to be compared with question 1 of Table 1).

It should be noted that all these findings are very valuable even because they were reported by patients themselves, who typically have much higher expectations than the surgeons. In addition, it has to be pointed out that when a patient forwarded several assessments over the years, we considered only the most negative evaluation, enabling the analysis to be more truthful, and free of possible bias.

We are conscious that this post-market study has some limitations due to the fact that the database was designed before 2007, when certain data were not thought to be so relevant and also because of a lack of awareness on how much these data would be important in the future. In this regard, the collection of some information, such as patients’ age, patients’ gender, BMI at surgery, causes of surgery (augmentation or reconstruction), and surgical techniques (eg, subglandular or submuscular position), would have made the results of this survey more precise and complete. In addition, CC Baker grade I, a normal health condition, and CC Baker grade II, a non-pathological condition in accordance with the majority of literature,^21,22^ have been listed among the possible complications. This discrepancy might have resulted in an overestimation of “any complication” cumulative incidence (Tables 5, 6). We are working on filling these gaps in the questionnaire in order to improve the quality of the further updates on this ongoing post-market safety analysis.

We also acknowledge that the follow-up time is not long enough to accurately estimate the risk of long-term complications. Nevertheless, we have reported all KM rates with the CIs, in order to provide an estimate of their precision at 5 years.

Another limitation of this study is that it is based on PROs, hence patient-related, with some complications that could, therefore, be missed or exaggerated. However, the importance of patient perspectives on disease impact is increasingly recognized by the scientific community. Moreover, PROs are generally unbiased and more closely reflect the real-world setting.

## CONCLUSIONS

The safety of POLYTECH breast implants has been demonstrated in clinical practice for more than 3 decades, and the IoE program helps the Company to constantly improve the PMS. The results of this analysis on a big cohort of patients validate the safety and performance of Memso breast implants at 5 years. In fact, Mesmo implants real-world data are very encouraging in terms of safety, low rate of adverse events, and overall patients’ satisfaction, even if the results need to be confirmed in the next years with a longer follow-up time.

## References

[1] Cronin TD, Gerow FJ. Augmentation mammaplasty: a new “natural feel” prosthesis. Transactions of the Third International Congress of Plastic Surgery. Amsterdam: Excerpta Medica Foundation; 1964. p. 41–49.

[2] Handel N, Guitierrez J. Long-term safety and efficacy of polyurethane foam-covered breast implants. Aesthet Surg J. 2006;26:265-274. doi: 10.1016/j.asj.2006.04.001 19338905

[3] Ashley FL . A new type of breast prosthesis. Preliminary report. Plast Reconstr Surg. 1970;45(5):421-424. doi: 10.1097/00006534-197005000-00001 5438186

[4] Perry D, Frame JD. The history and development of breast implants. Ann R Coll Surg Engl. 2020;102(7):478-482. doi: 10.1308/rcsann.2020.0003 31964154 PMC7450417

[5] Namnoum JD, Largent J, Kaplan HM, et al. Primary breast augmentation clinical trial outcomes stratified by surgical incision, anatomical placement and implant device type. J Plast Reconstr Aesthet Surg. 2013;66(9):1165-1172. doi: 10.1016/j.bjps.2013.04.046 23664574

[6] Aesthetic Plastic Surgery National Databank Statistics 2020. Aesthet Surg J. 2021;41(Suppl 2):1-16. doi: 10.1093/asj/sjab178 33880491

[7] Atlan M, Bigerelle M, Larreta-Garde V, et al. Characterization of breast implant surfaces, shapes, and biomechanics: a comparison of high cohesive anatomically shaped textured silicone, breast implants from three different manufacturers. *Aesth Plast Surg.* 2016;40(1):89-97. doi: 10.1007/s00266-015-0603-8 26746882

[8] Spear SL, Murphy DK. Natrelle round silicone breast implants: core study results at 10 years. Plast Reconstr Surg. 2014;133(6):1354-1361. doi: 10.1097/PRS.0000000000000021 24867717 PMC4819531

[9] Caplin DA . Indications for the use of MemoryShape breast implants in aesthetic and reconstructive breast surgery: long-term clinical outcomes of shaped versus round silicone breast implants. Plast Reconstr Surg. 2014;134(3 Suppl):27S-37S. doi: 10.1097/PRS.0000000000000609 25158767

[10] Calobrace MB, Stevens WG, Capizzi PJ, et al. Risk factor analysis for capsular contracture: a 10-year Sientra study using round, smooth, and textured implants for breast augmentation. Plast Reconstr Surg. 2018;141(4S):20S-28S. doi: 10.1097/PRS.0000000000004351 29595715

[11] Maxwell GP, Van Natta BW, Bengtson BP, Murphy DK. Ten-year results from the Natrelle 410 anatomical form-stable silicone breast implant core study. Aesthet Surg J. 2015;35(2):145-155. doi: 10.1093/asj/sju084 25717116 PMC4399443

[12] Derby BM, Codner MA. Textured silicone breast implant use in primary augmentation: core data update and review. Plast Reconstr Surg. 2015;135(1):113-124. doi: 10.1097/PRS.0000000000000832 25539301

[13] Spear SL, Baker JL, Jr. Classification of capsular contracture after prosthetic breast reconstruction. Plast Reconstr Surg. 1995;96(5):1119-1123; discussion 1124. 7568488

[14] Pompei S, Arelli F, Labardi L, et al. Polyurethane implants in 2-stage breast reconstruction: 9-year clinical experience. Aesthet Surg J. 2016;37(2):171-176. doi: 10.1093/asj/sjw183 27940908

[15] ISO International Organization for Standardization. ISO 14607: 2018—non-active surgical implants—mammary implants—particular requirements. Accessed November 13, 2019. https://www.iso.org/standard/63973.html

[16] Barr S, Hill EW, Bayat A. Functional biocompatibility testing of silicone breast implants and a novel classification system based on surface roughness. J Mech Behav Biomed Mater. 2017;75:75-81. doi: 10.1016/j.jmbbm.2017.06.030 28697402

[17] Doward LC, Gnanasakthy A, Baker MG. Patient reported outcomes: looking beyond the label claim. Health Qual Life Outcomes. 2010;8(1):89-97. doi: 10.1186/1477-7525-8-89 20727176 PMC2936442

[18] US Food and Drug Administration. Guidance for Industry: Patient-Reported Outcome Measures: Use in Medical Product Development to Support Labeling Claims. US Food and Drug Administration; 2009.

[19] European Medicines Agency Committee for Medicinal Products for Human Use. Appendix 2 to the Guideline on the Evaluation of Anticancer Medicinal Products in Man: The Use of Patient-Reported Outcome (PRO) Measures in Oncology Studies EMA/CHMP/292464/2014. European Medicines Agency; 2016.

[20] Adams WP, Jr . The process of breast augmentation: four sequential steps for optimizing outcomes for patients. Plast Reconstr Surg. 2008;122(6):1892-1900. doi: 10.1097/PRS.0b013e31818d20ec 19050543

[21] Government of Canada. Summary Basis of Decision (SBD), Natrelle™ Highly Cohesive Silicone-Filled Breast Implants Allergan Medical. P040046. Application Number: 88573, Licence Number: 72262, Health Canada. Date Issued 2008/07/18.

[22] Government of Canada. Summary Basis of Decision (SBD) Mentor Memorygel™ CPG Breast Implants Cohesive III Mentor Medical Systems B.V. P060028. Application Number: 86150, Licence Number: 72270, Health Canada. Date Issued 2010/09/23.

[23] DeCoster RC, Lynch EB, Bonaroti AR, et al. Breast implant-associated anaplastic large cell lymphoma: an evidence-based systematic review. Ann Surg. 2021;273(3):449-458. doi: 10.1097/SLA.0000000000004365 33234792

[24] Lukavsky RJ, Couto RA, Adams WP. Is breast implant-associated anaplastic large cell lymphoma better classified as a lymphoproliferative disorder and how surgeons reduce risk? Clin Plast Surg. 2021;48(1):71-77. doi: 10.1016/j.cps.2020.08.002 33220906

[25] Khanna J, Mosher M, Whidden P, et al. Reoperation rate after primary augmentation with smooth, textured, high fill, cohesive, round breast implants (RANBI-I Study). Aesthet Surg J. 2019;39(12):1342-1349. doi: 10.1093/asj/sjy289 30383228 PMC6853655

[26] Stevens WG, Calobrace MB, Alizadeh K, et al. Ten-year core study data for Sientra’s Food and Drug Administration-approved round and shaped breast implants with cohesive silicone gel. Plast Reconstr Surg. 2018;141(4S):7S-19S. doi: 10.1097/PRS.0000000000004350 29595714

[27] Marques M, Brown SA, Oliveira I, et al. Long-term follow-up of breast capsule contracture rates in cosmetic and reconstructive cases. Plast Reconstr Surg. 2010;126(3):769-778. doi: 10.1097/PRS.0b013e3181e5f7bf 20463624

[28] Ho AL, Bovill ES, Macadam SA, et al. Postmastectomy radiation therapy after immediate two-stage tissue expander/implant breast reconstruction: a University of British Columbia perspective. Plast Reconstr Surg. 2014;134(1):1e-10e. doi: 10.1097/PRS.0000000000000292 25028850

[29] Tallet AV, Salem N, Moutardier V, et al. Radiotherapy and immediate two-stage breast reconstruction with a tissue expander and implant: complications and esthetic results. Int J Radiat Oncol Biol Phys. 2003;57(1):136-142. doi: 10.1016/s0360-3016(03)00526-1 12909226

[30] Loreti A, Siri G, De Carli M, et al. Immediate breast reconstruction after mastectomy with polyurethane implants versus textured implants: a retrospective study with focus on capsular contracture. The Breast. 2020;54:127-132. doi: 10.1016/j.breast.2020.09.009 33010626 PMC7529839

[31] Clarke-Pearson EM, Lin AM, Hertl C, et al. Revision in implant-based breast reconstruction: how does direct-to-implant measure up? Plast Reconstr Surg. 2016;137(6):1690-1699. doi: 10.1097/PRS.0000000000002173 27219225

[32] Sue GR, Sun BJ, Lee GK. Complications after two-stage expander implant breast reconstruction requiring reoperation: a critical analysis of outcomes. Ann Plast Surg. 2018;80(5S):S292-S294. doi: 10.1097/SAP.0000000000001382 29489547

[33] Hammond DC, Canady JW, Love TR, et al. Mentor contour profile gel implants: clinical outcomes at 10 years. Plast Reconstr Surg. 2017;140(6):1142-1150. doi: 10.1097/PRS.0000000000003846 29176413

[34] Montemurro P, Cheema M, Hedén P, et al. Do not fear implant’s shape: a single surgeon’s experience of over 1200 round and shaped textured implants in primary breast augmentation. Aesthet Surg J. 2018;38(3):254-261. doi: 10.1093/asj/sjx145 29106482

[35] Montemurro P, Tay VKS. Transitioning from conventional textured to nanotextured breast implants: our early experience and modifications for optimal breast augmentation. Aesthet Surg J. 2021;41(2):189-195. doi: 10.1093/asj/sjaa169 32582953

[36] Montemurro P, Cheema M, Khoda B, et al. Two-person screening of mental well-being before primary breast augmentation: can we do more? J Plast Reconstr Aesthet Surg. 2021;74(1):152-159. doi: 10.1016/j.bjps.2020.08.125 33082077

[37] Pusic AL, Matros E, Fine N, et al. Patient-reported outcomes 1 year after immediate breast reconstruction: results of the Mastectomy Reconstruction Outcomes Consortium Study. J Clin Oncol. 2017;35(22):2499-2506. doi: 10.1200/JCO.2016.69.9561 28346808 PMC5536162

[38] Ng S, Pusic A, Parker E, et al. Patient-reported outcome measures for breast implant surgery: a pilot study. Aesthet Surg J. 2019;39(8):NP314-NP321. doi: 10.1093/asj/sjz023 30783646

